# TSH Isoforms: About a Case of Hypothyroidism in a Down's Syndrome Young Adult

**DOI:** 10.4061/2010/703978

**Published:** 2010-07-14

**Authors:** Anne-Sophie Gauchez, Magali Pizzo, Dany Alcaraz-Galvain, Karim Chikh, Jacques Orgiazzi, Georg Brabant, Catherine Ronin, Anne Charrié

**Affiliations:** ^1^Laboratoire du Service de Médecine Nucléaire, Centre Hospitalier de Chambéry, 73000 Chambéry, France; ^2^Département de Biologie Intégrée, Centre Hospitalier et Universitaire de Grenoble, F38043 Grenoble Cedex 9, France; ^3^Société Française de Médecine Nucléaire, Groupe de Biologie Spécialisée, Centre Antoine Béclère, 45 rue des Saints Pères, 75270 Paris, France; ^4^INSERM U877, 38000 Grenoble, France; ^5^Neurodiag, UMR 6149, CNRS et Université de Provence, 13331 Marseille, France; ^6^Unité Fonctionnelle Endocrinologie, Nutrition, Métabolisme, Département de Biologie, Hospices Civils de Lyon, Centre Hospitalier Lyon sud, 69495 Pierre-Bénite, France; ^7^INSERM U590, Lyon 69008, France; ^8^Service Endocrinologie, Diabète, Nutrition, Hospices Civils de Lyon, Centre Hospitalier Lyon Sud, 69495 Pierre-Bénite, France; ^9^Department of Endocrinology, The Christie, Manchester M20 4BX, UK; ^10^Faculté de Médecine Lyon Sud-Charles Mérieux, Université de Lyon, 69622 Lyon, France

## Abstract

*Background*. For unknown reasons, the prevalence of thyroid autoimmune disorders is higher in patients with Down's syndrome than in the general population. The present case strongly supports a recent evaluation of propagating screening for thyroid disease in this group of patients to assure early diagnosis of hypothyroidism. *Methods*. In a 25-year-old man diagnosed with Down's syndrome, clinical manifestations of hypothyroidism were lacking, but profound biochemical abnormalities were found with particularly high levels of thyroid stimulating hormone (TSH). Antigenic properties of TSH were characterized using a panel of anti-TSH antibodies. *Results*. Technical problems not infrequently associated with TSH measurements are convincingly ruled out. Antigenic characterization of the patient's circulating TSH revealed circulating forms of TSH different from pituitary TSH which closely resembled TSH recombinant human hormone. *Conclusions*. It appears counterintuitive that the bioactivity of TSH decreases in the hypothyroid state as higher bioactivity of TSH is anticipated in hypothyroidism promoted by an increased hypothalamic TRH drive. In contrast, diminished negative thyroid hormone feedback will enhance posttranslational glycosylation of TSH subunits and increase sialylation of the carbohydrate side chains. Both exert a negative effect on TSH bioactivity, only compensated by the very high levels of the hormone as in the present case.

## 1. Introduction

For currently unknown reasons, the prevalence of thyroid autoimmune disorders (AITD) is higher in patients with Down's syndrome than in the general population which is observed in up to 3% of children. Most frequently, this is associated with subclinical hypothyroidism. Systematic screening for thyroid disease has been recently advocated in this group of patients [1] to insure early diagnosis of hypothyroidism.

## 2. Patient and Methods

### 2.1. Patient

In a 25-year-old man with Down's syndrome diagnosed at one month of life because of delayed growth, a thyroid biochemical workup (thyrotropin, TSH; free thyroxin, FT4 and free triiodothyronine, FT3) was performed on the request of his psychiatrist. The patient had no apparent clinical evidence of a thyroid disorder and was never tested before for a thyroid disorder although he had a positive family history including spontaneous hypothyroidism in his grandmother and Graves' disease in his mother. The young man was not on medication at the time of blood sampling; his weight was 56 kg and his pulse 60/min.

The following thyroid test results were obtained: TSH: 1392 mIU/L (reference value: 0.25–4.0 mIU/L), FT4: 0.66 pmol/L (reference value: 8.2–18 pmol/L), and FT3: 1.14 pmol/L (reference value: 4.2–8.3 pmol/L) (Table 1). Antithyroid peroxidase, antithyroglobulin, and anti-TSH receptor antibodies (TRab) were determined, as well as the biological activity of the latter. Additional assays included total cholesterol, total testosterone, luteinizing hormone (LH), follicle-stimulating hormone (FSH), prolactin, and *α*-subunit. Results are summarized in Table 1. TRAbs were assayed at the time when TSH was 1200–1540 mIU/L. The result of the TRAK assay was 216 U/L. The assay of the biological activity of TRAb indicated that both stimulating and blocking activities were detectable. Stimulating activity was 243% of control, and blocking amounted to 81%, a very high value. 

Because of the discrepancy between the clinical and biological status and the unusual elevation of the TSH level, a second blood sample was tested for TSH, FT4, and FT3 with qualitatively comparable results. In order to exclude a potential interference from heterophilic antibodies, the serum was pretreated with Heterophilic Binding Tubes (HBT, Scantibodies, Villebon-Sur-Yvette, France). Unaltered TSH, FT4, and FT3 levels argued against any interference. We further excluded antithyroid hormone (anti-T3, anti-T4) and anti-TSH autoantibodies (Table 1). Radioelectrophoresis showed no abnormality in binding of thyroxin to plasma proteins.

Subsequently, the patient was treated with 50 *μ*g levothyroxine (Levothyrox) per day. He complained of fatigue and a weight loss of 2 kg after 15 days of treatment with an unchanged pulse rate of 56 bpm. Nuclear Magnetic Resonance imaging of the adenohypophysis excluded a pituitary adenoma with a rather small gland. Three months later, the patient had no clinical complaints; his heart rate was stable at 60 bpm. Levothyrox dose was increased to 100 *μ*g/d. TSH level had dropped to 10.8 mIU/L, and FT4 as well as FT3 increased to 8.12 pmol/L and 3.9 pmol/L, respectively. Total cholesterol was 4.8 mol/L, alpha-subunit and prolactin levels were within the reference range. To rule out any interference of the very high initial concentrations of endogenous TSH on the radioreceptor as well as of the biological assays, TRAb levels were retested under levothyroxine treatment. With an endogenous TSH concentration of 10,8 mUI/L, TRAK levels were 384 U/L; the stimulating activity was 189%; and the blocking activity was 77%, both not different from the previously obtained results.

### 2.2. Assay Methods

TSH assay was performed using three immunoradiometric kits (Cis Bio International, IBA, Gif-sur Yvette, France; Roche Diagnostic, Meylan, France and Beckman-Coulter-Immunotech, Marseille, France). FT4 and FT3 were measured using the Cis Bio International, IBA, and Beckman-Coulter-Immunotech radioimmunoassays (RIA); antithyroid peroxidase, antithyroglobulin, and anti-TSHR antibodies were measured with the corresponding RIA kit (Brahms-Diagnostica, Berlin, Germany); prolactin, LH, FSH; and *α*-subunit were measured with the RIA kits from Beckman-Coulter-Immunotech; testosterone was measured with the Cis Bio International RIA. Total cholesterol was measured using an enzymatic method (Olympus, France). Anti-T4, anti-T3, and anti-TSH antibodies were assessed with in-house RIA. The stimulating and blocking biological activities of anti-TSH antibodies were assayed as described previously by Madec et al. [2, 3], and cAMP was determined by RIA (Beckman-Coulter-Immunotech). Radioelectrophoresis was performed according to an in-house technique: serum incubated with ^125^I thyroxin (7.4 kBq/*μ*L) was analyzed by agarose gel electrophoresis with bromophenol blue staining of proteins. The relative distribution of radioactive T4 among thyroxin binding protein (TBG), albumin, transthyretin, and T4-binding immunoglobulin when present is calculated as a function of the total radioactivity added to the serum. Reference values were established on 40 sera from euthyroid normal subjects.

## 3. Immunochemical Characterization of TSH

**Figure 1:**
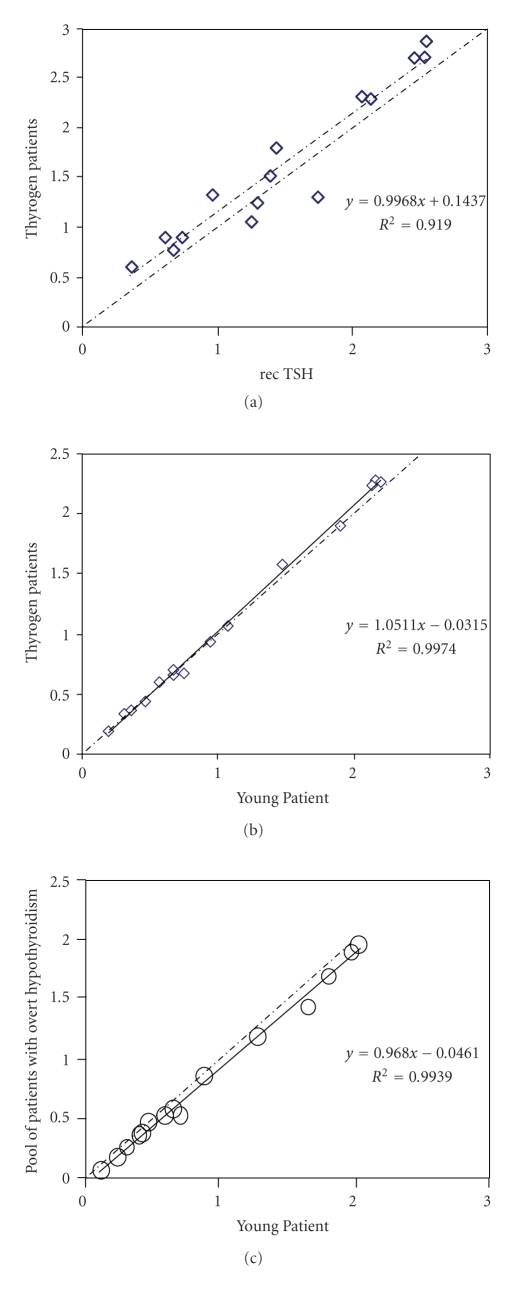
(a) Hormone circulating in cancer patients injected with Thyrogen: pool of 4 patients, TSH = 311 mIU/L (Thyrogen patients) versus recTSH. (b) Thyrogen patients versus young patient. (c) Pool of patients with overt hypothyroidism (TSH approx. 177 mIU/L) versus young patient.

Earlier studies have shown that primary hypothyroidism resulted in a drastic alteration of TSH epitope expression [4], and that changes in TSH immunoreactivity could be detected with anti-TSH antibodies. Indeed, both sialylation and fucosylation could increase antibody recognition [4]. Therefore, the carbohydrate structure of the patient's TSH was studied and compared to that of recombinant TSH in accordance to previous work [5]. As shown in Figure 1(a), we first performed comparative epitope mapping of recombinant TSH (recTSH) to the hormone circulating in cancer patients injected with Thyrogen (pool of 4 patients, TSH = 311 mIU/L). A panel of 10 monoclonal antibodies targeting 3 main antigenic clusters was used in the best 15 distinct formats reported earlier [4]. Figure 1(a) shows that recTSH and the circulating drug behaved quite similarly, indicating that antibodies recognized the same forms in both samples with equal efficiency. When the circulating TSH of our patient and of thyroid cancer patients were compared, immunoreactivities of both endogenous TSH and Thyrogen (Figure 1(b)) were plotted on the same line with a slope of 1.0511 in all formats, with *r* ^2^ close to 1. We also compared the immunoreactivities of the patient's circulating TSH to that of a pool of patients with overt hypothyroidism (average TSH level: 177 mIU/L) with undetectable thyroid hormones and no thyroid autoantibodies.  Figure 1(c) shows that the two blood samples were similar with a slope value of 0.968 and *r* ^2^ value close to 1. Therefore, the TSH of the patient clearly appeared to have similar glycoepitopes as that from patients with primary hypothyroidism.

However, from previous studies using identical immunotyping we know that pituitary TSH and serum TSH from patients with primary hypothyroidism deviate with a slope value (standard deviation) of 2.124 (0.001), with *r* ^2^ = 0.817 [6]. This finding supports the notion that TSH of our patient was definitely abnormal as antibodies were able to bind to these hormonal glycoforms in the same way as to recombinant TSH and much more efficiently than to pituitary normal TSH. 

In summary, these data indicate that the high circulating TSH levels in our patient were associated with profound changes in hormone immunoreactivity, suggesting alterations in the glycosylation pattern similar to those found in overt primary hypothyroidism. Using TSH preparations modified for sialylation and/or fucosylation, we were able to ascribe these structural changes to alterations in terminal and/or internal glycosylation occurring in primary hypothyroidism [4]. These modifications, independently or in combination, alter the expression of most of the TSH epitopes, presumably through a change in the *β*-subunit conformation.

## 4. Discussion

The prevalence of overt thyroid diseases in children with Down's syndrome is about 3% [7]. However, the frequency of serum TSH > 10 mIU/L in the same population varies from 13 to 50%. 

In most of the cases, the elevation of TSH is due to an autoimmune dysfunction with the presence of anti-thyroid antibodies. However, autoimmune subclinical hypothyroidism must be distinguished from defects in the biological activity of the TSH molecule. TSH bioactivity in the plasma of Down's syndrome children is normal indicating that subclinical hypothyroidism in these patients results from primary thyroid dysfunction [8].

In the present case, the unusually high elevation of the serum TSH concentration and its combination with the presence of a high level of TRAb, particularly of the blocking type, raise several questions pertaining to (i) the duration and the intensity of the primary thyroid insufficiency, (ii) the role of the blocking TRAb in the mechanism of hypothyroidism, and (iii) the possibility of changes in the metabolic clearance and possibly in the immune-recognition of the circulating TSH due to alterations in the glycosylation pattern of the molecule.

Very high levels of TSH are usually observed in long-lasting and severe hypothyroidism. In this case, however, clinical manifestations, if any, were very mild, although some improvement was noticed on thyroxine substitution. Such discrepancy between clinical and biological status, although unexplained, is not uncommon. The lack of pituitary hypertrophy is more surprising if a case of long-standing juvenile hypothyroidism is assumed. This supports the alternative hypothesis that hypothyroidism, although severe, was of recent onset, possibly related to the presence of blocking TRAb. 

Pituitary TSH is synthesized and secreted as a variable mixture of a dozen of different glycoforms. This polymorphism influences the metabolic clearance rate and bioactivity of TSH. Indeed, sialylation of the hormone molecule prolongs its blood half-life by preventing its liver uptake. On the other hand, several studies have shown that the bioactivity of TSH is enhanced or reduced depending on the pathophysiological conditions [9–11]. TSH, about 30 kDa, belongs to the glycoprotein hormone family. These glycoproteins share a high degree of glycosylation and are heterodimers consisting of a common *α*-subunit and a unique *β*-subunit, which confers the biological and immunological specificity to each hormone. TSH contains 3 N-glycan chains of complex type constituting between 15 to 25% of its molecular mass. The terminal glycosylation, the inner fucosylation, and the degree of connection are three parameters of heterogeneity responsible for the natural polymorphism of this hormone.

This paper shows that TSH expresses three of its main epitopes to a different extent according to the nature of its glycosylation. The antibodies, which readily recognize the glycoforms in the pituitary calibrator, are also able to recognize the same glycoforms present in serum TSH (or the recombinant hormone), but present in small quantity because of the metabolic clearance. This is the basis of the currently marketed measurement systems since all assays are calibrated against the 2nd International Reference Preparation (IRP) of human pituitary TSH, 80/558. Since these forms are decreasing in the circulation and the more sialylated forms of TSH increase as the hypothyroidism develops, it can be assumed that monoclonal antibodies bind less to normally expected and more to diseased forms of TSH for which they display increased efficiency. This differential recognition may in turn affect TSH measurement, leading to a biased follow up of the hormone level, especially under T4 treatment when TSH levels could be normalized. Despite the limitations, we were able to show that TSH of this patient is structurally abnormal and virtually identical to hypothyroid TSH, reflecting probably a long-lived TSH with very low biological activity.

##  Conflicts of Interest

None of the authors have accepted any funding or support from an organization that may in any way gain or lose financially from the results of our study. None of the authors have been employed by any organization that may in any way gain or lose financially from the results of our study.

## Figures and Tables

**Table 1:** Control of initial thyroid parameters TSH, FT3, and FT4 by different assays and additional biological parameters.

	Patient's results	Reference values
TSH	1392^1^	0.25–4 mIU/L
	1200^2^	0.27–4.2 mIU/L
	1540^3∗^	0.29–3.8 mIU/L

FT4	0.66^4^	8.2–18 pmol/L
	1.6^5^	11.5–23 pmol/L
	1.3^5∗^	13–22.6 pmol/L

FT3	1.14^4^	4.2–8.3 pmol/L
	1.7^5^	2.5–5.8 pmol/L
	1.9^5∗^	2.8–5.3 pmol/L

Anti-thyroid peroxidase antibodies (AbTPO)^ 6^	4047	<60 kU/L

Thyroglobulin (Tg)^ 1^	< 0.7	<50 *μ*g/L

Anti-thyroglobulin antibodies (AbTg)^ 6^	198	<60 kU/L

Anti-TSH receptor antibodies (TSHR)^ 6^	216	<1.0 U/L

Blocking anti-TSHR^ 7^	81	<10%

Stimulating anti-TSHR^ 7^	243	100%

Anti-T3 antibodies^ 7^	4.9	<7.9%

Anti-T4 antibodies^ 7^	3.7	<7%

Anti-TSH antibodies^ 7^	13	<18%

Total cholesterol^8^	9.3	4.5–6.0 mmol/L

Total Testosterone^1^	11.3	8.2–34.6 nmol/L

Alpha-subunits^3^	2.8	<0.8 IU/L
Prolactin^3^	1009	30–545 mIU/L

Luteinizing hormone (LH)^ 3^	4.6	1.8–8.4 IU/L

Follicle-stimulating hormone (FSH)^ 3^	18.3	2.2–10 IU/L

Radioelectrophoresis:		
(i) Thyroxin-Binding Protein	69.7	63.6–81.2%
(ii) Albumin	6.0	2.9–9.7%
(iii) Transthyretin	23.8	12.5–29.7%
(iv) Immunoglobulins	0.5	<2.0%

^1^CisBio, ^2^Modular Roche Diagnostic, ^3^IRMA Beckman Coulter, ^4^RIA lisophase Cis Bio, ^5^RIA Beckman Coulter, ^6^Brahms, ^7^in-house assay, Biological Center Lyon Sud, ^8^Olympus. *Pretreatment with “Heterophilic Blocking Tube” scantibodies.
